# Does aluminum exposure affect cognitive function? a comparative cross-sectional study

**DOI:** 10.1371/journal.pone.0246560

**Published:** 2021-02-16

**Authors:** Tao Zhang, Fan He, Shangtong Lin, Xinyi Wang, Fudong Li, Yujia Zhai, Xue Gu, Mengna Wu, Junfen Lin

**Affiliations:** 1 Department of Public Health Surveillance and Advisory, Zhejiang Provincial Center for Disease Control and Prevention, Hangzhou, Zhejiang, China; 2 Office, Cangnan Center for Disease Control and Prevention, Wenzhou, Zhejiang, China; Government College University Faisalabad, Pakistan, PAKISTAN

## Abstract

**Objectives:**

This study assessed the cognitive function of aluminum-exposed participants from an alum mining zone, compared them with unexposed subjects, and aimed to elucidate the effect of aluminum exposure on cognition.

**Design:**

This was a comparative cross-sectional study. Univariate analyses were used to assess the differences between the aluminum-exposed and unexposed groups. Binary logistic regression models were applied to analyze the effect of aluminum exposure.

**Setting:**

The aluminum-exposed participants were included from an alum mining zone and the unexposed subjects were residents from another district without alum-mine-related factories.

**Participants:**

We included 539 aluminum-exposed participants (254 men, 285 women) and 1720 unexposed participants (692 men, 1028 women).

**Results:**

The mean cognition score on Mini-Mental State Examination was 21.34 (± 6.81) for aluminum-exposed participants. The exposed group had 6.77 times (95% confidence interval, 5.09–9.00) more risk of cognitive impairment than the unexposed group, after adjusting for age, sex, and educational level. No statistically significant association was found between exposure duration and cognition.

**Conclusions:**

This study demonstrated a significant association between aluminum exposure and lower cognitive function.

## Introduction

Aluminum is the most abundant metal in the Earth’s crust. It has been widely used in cooking utensils and in recent decades, has been found in foods in China, such as deep-fried dough sticks [1, 2].

Aluminum is recognized as a catalyst for Alzheimer’s disease; in the absence of brain-burdening aluminum, Alzheimer’s disease is not an inevitable consequence of aging [3]. Since the 1960s, when Alzheimer’s-like neuronal lesions were found in rabbits that had been treated with a compound containing aluminum [4], the causal relationship between aluminum and dementia has been the subject of ongoing research [5–7].

The effect of aluminum on cognition is partially owning to its interaction with tau proteins. Aluminum may promote the development of neurofibrillary tangles (NFTs) by promoting phosphorylation of tau proteins [8]. There is also evidence that aluminum affects amyloid-β (Aβ) proteins by promoting the production of Aβ aggregates and inhibiting their degradation [9–12]. Aluminum can also upregulate the expression for amyloid-β precursor protein (APP) gene and other stress-response genes in human neural cells [13]. It has also been reported that aluminum can affect neurotransmission. Because aluminum has the ability to block the formation of calcium-permeable ion channels mediated by Aβ, it can inhibit the increase in calcium influx induced by neurotrophic factors such as the brain derived neurotrophic factor [14–17].

Few epidemiological studies have focused on the effects of occupational aluminum exposure on cognition. Cognitive decline has been found in smelting workers in aluminum factories [18–20]. Iregren et al [21] reported that aluminum welders showed reduced performance in four motor function tests and one pegboard test, however the reduction was not significant. Sim et al [22] found no significant effects in aluminum potroom workers using objective measures of neurological function. However, these studies were all limited by small sample sizes.

Alum is a natural, common, aluminum-containing compound and is a raw material used for aluminum production. A huge alum mine in southeastern China was founded about one hundred years ago, and a residential zone was developed around the mine. Before 2004, there were also many factories engaged in bauxite mining and processing, and many local residents worked in these factories. High dust concentration was a common problem in many alum mines in China before the 1990s [23], and serious health problems among miners, such as silicosis, were widely reported.

Our study enrolled workers from the alum mine, assessed their cognitive function, compared them with aluminum-unexposed participants, and aimed to demonstrate the effect of aluminum exposure on cognition. We also hypothesized that the risk of cognitive impairment increased with occupational exposure duration.

## Materials and methods

Data was obtained from a public health surveillance project aimed at exploring health problems among elderly people in Zhejiang [24]. The project was conducted in all 11 cities in Zhejiang since 2014, and each city chose at least one county to recruit a minimum of 1000 permanent residents aged 60 years and older. The counties were chosen according to local disease patterns, exposure to certain risk factors, population stability, quality of death and disease registries, local commitment, and the capacity of staff. In Wenzhou, the surveillance population was extended to adults aged 18 years and older, and Cangnan, where the alum mine is located, was the chosen surveillance county. Face-to-face interviews were completed by well-trained interviewers with a questionnaire that included sociodemographic information, work experience, cognition data, and current medical history. The study was approved by the Ethics Committee of Zhejiang Provincial Center for Disease Control and Prevention and conducted in accordance with the principles of the Declaration of Helsinki of the World Medical Association. Written informed consent was obtained from each participant.

In our study, we selected participants from Cangnan and Yuhuan who were surveyed in 2016. Local residents of Cangnan with occupational exposure to dust (self-reported; only alum miners were occupationally exposed to dust in Cangnan) or work experience in the alum-mine-related factories (self-reported) were included in the aluminum-exposed group. To avoid the influence of unobserved confounders, unexposed participants were selected from Yuhuan, a county without alum-mine-related factories. Both Yuhuan and Cangnan are coastal, and the distance between the two counties is about 100 kilometers. Among the participants, there were some similarities in diets and living conditions between the two areas. We selected unexposed participants from Yuhuan instead of Cangnan because of the history of severe environmental pollution in Cangnan, which could affect cognition. In our study, the miners were local residents, meaning they were subject to both environmental and occupational exposure.

Cognitive function was measured by the Mini-Mental State Examination (MMSE), which includes 30 items. The MMSE is brief and easy to administer to elderly people and those with low education levels. It has become one of the most commonly used screening tools to evaluate cognitive function in epidemiological studies with large sample sizes [25, 26]. The maximum score on the MMSE is 30, and higher scores indicate better cognitive function. A battery of education-specific cut-off scores for cognitive impairment was used: 17/18 for illiteracy, 20/21 for people with primary education, and 24/25 for people with a higher than primary education [24]. MMSE subscores are calculated by grouping various items of the MMSE by domain: orientation to time (0–5 points possible), orientation to place (0–5), registration (0–3), recall (0–3), attention and calculation (0–5), language (0–8), and figure (0–1). More details about the MMSE scale have been described elsewhere [27].

Sociodemographic factors included age, sex, educational level, and economic status. Alum-mine-related work experience included the specific job type and the time when one began and left the job. Other factors included hypertension, hyperlipidemia, diabetes mellitus, stroke, acute myocardial infarction, tumor (either malignant or benign), severe head trauma, smoking status, and alcohol consumption status.

We compared sociodemographic and other characteristics between the two groups, using Welch’s t-test (for continuous variables) and Fisher’s exact test (for categorical variables). Binary logistic regression models were applied to analyze the effect of occupational exposure duration in the alum mines. Model 1 included aluminum exposure status, age, sex, and education; Model 2 included hypertension, diabetes, tumor, smoking, and alcohol consumption based on model 1. All statistical analyses in this study were performed using R version 3.5.1 and SAS version 9.2 (SAS Institute, Cary, NC, USA), and a two-tailed p-value <0.05 was considered statistically significant.

## Results

The mean age of aluminum-exposed participants was 57.3 years, 13 years younger than that of unexposed group. The proportion of illiteracy among the exposed and unexposed groups was 44.9% and 68.8%, respectively. The proportions of hypertension, diabetes, and tumor in the exposed group were significantly lower than those in the unexposed group. The aluminum-exposed group performed worse than the unexposed group on the MMSE, with a lower mean score and a higher proportion of that group having cognitive impairment. More details are shown in Table 1.

**Table 1:** Characteristics of the aluminum-exposed and unexposed participants.

Characteristics		Overall	Unexposed group	Exposed group	*P*
*n*		2259	1720	539	
Age (years, mean (SD))		67.0 (11.0)	70.0 (7. 8)	57.3 (13.7)	<0.001
Sex	Male	946 (41.9)	692 (40.2)	254 (47.1)	0.005
	Female	1313 (58.1)	1028 (59.8)	285 (52.9)	
Education (%)	Illiteracy	1426 (63.1)	1184 (68.8)	242 (44.9)	<0.001
	Primary school	660 (29.2)	460 (26.7)	200 (37.1)	
	Middle school and higher	173 (7.7)	76 (4.4)	97 (18.0)	
Hypertension (%)	No	1193 (52.8)	810 (47.1)	383 (71.1)	<0.001
	Yes	1066 (47.2)	910 (52.9)	156 (28.9)	
Hyperlipidemia (%)	No	2136 (94.6)	1625 (94.5)	511 (94.8)	0.828
	Yes	123 (5.4)	95 (5.5)	28 (5.2)	
Diabetes (%)	No	1987 (88.0)	1477 (85.9)	510 (94.6)	<0.001
	Yes	272 (12.0)	243 (14.1)	29 (5.4)	
Tumor (%)	No	2208 (97.7)	1670 (97.1)	538 (99.8)	<0.001
	Yes	51 (2.3)	50 (2.9)	1 (0.2)	
Smoking (%)	Never smokers	1835 (81.2)	1395 (81.1)	440 (81.6)	0.075
	Current smokers	305 (13.5)	225 (13.1)	80 (14.8)	
	Ex-smokers	119 (5.3)	100 (5.8)	19 (3.5)	
Alcohol consumption (%)	Never drinkers	1380 (61.1)	1322 (76.9)	58 (10.8)	<0.001
	Current drinkers	65 (2.9)	56 (3.3)	9 (1.7)	
	Ex-drinkers	814 (36.0)	342 (19.9)	472 (87.6)	
MMSE score (mean (sd))		22.56 (5.85)	22.95 (5.46)	21.34 (6.81)	<0.001
Cognitive impairment	No	1779 (78.8)	1414 (82.2)	365 (67.7)	<0.001
	Yes	480 (21.2)	306 (17.8)	174 (32.3)	

### Aluminum exposure and cognition

We used two logistic regression models to detect the effect of aluminum exposure on cognitive impairment. The aluminum-exposed group had 6.77 times more risk of cognitive impairment than the unexposed group, adjusted for age, sex, and educational level (Model 1). The prevalence odds ratio (POR) remained high when adjusted for more covariates (Model 2). More details are shown in Table 2.

**Table 2:** Associations between cognitive impairment and multiple factors.

	Model 1			Model 2		
	*POR*	95% *CI*	*P*	*POR*	95% *CI*	*P*
Aluminum exposure	**6.77**	5.09–9.00	<0.001	**8.21**	5.55–12.14	<0.001
Age	1.09	1.08–1.11	<0.001	1.09	1.08–1.11	<0.001
Female	1.53	1.19–1.98	0.001	1.21	0.89–1.64	0.216
Education						0.217
Illiteracy	reference			reference		
Primary school	0.78	0.58–1.04	0.088	0.77	0.58–1.04	0.090
Middle school and higher	1.00	0.60–1.64	0.984	0.98	0.59–1.63	0.936
Hypertension				0.93	0.73–1.18	0.541
Diabetes				0.80	0.56–1.15	0.223
Tumor				1.38	0.66–2.89	0.388
Smoking						0.030
Never smokers				reference		
Current smokers				0.64	0.42–0.98	0.039
Ex-smokers				0.49	0.24–0.99	0.047
Alcohol consumption						0.260
Never drinkers				reference		
Current drinkers				1.02	0.47–2.25	0.952
Ex-drinkers				0.74	0.52–1.07	0.109

### Occupational exposure duration and cognition

The mean exposure duration was 13.2 (± 11.3) years in the exposed group. We found no statistically significant association between occupational exposure duration and cognition after analysis by logistic regression (covariates included age, sex, and education; *p* = 0.232).

## Discussion

This study demonstrated a correlation between aluminum exposure and lower cognition test scores and increased risk of cognitive impairment. Aluminum-exposed subjects had over six times more risk of cognitive impairment than unexposed subjects.

Most aluminum-exposed subjects in our study reported a history of dust inhalation when working in the alum mine, and had been living in the area surrounding the alum mine for decades. The working environment of alum mine workers before 2000 was problematic. According to He et al [28], the incidence of silicosis in alum mine workers increased from 1970 to 1988, and in newly diagnosed patients, the average exposure duration from working in the mine was 17.87 years. Also, the air pollution resulting from aluminum production in the area cannot be ignored. In this study, the association between the risk of cognitive impairment and exposure duration was not statistically significant. A possible reason for this was that the effect of occupational exposure duration was masked by the effect of environmental exposure.

Our study had a larger sample size than did previous studies. In contrast to the studies by Iregren et al [21] and Sim et al [22], our study showed a statistically significant association between aluminum exposure and cognitive impairment.

Aluminum is neurotoxic and among the most studied metals, with many studies investigating its relationship with dementia [29–31]. Experimental studies in rats and mice have shown that aluminum can accumulate in the cerebral cortex, hippocampus, and cerebellum [32]. The PAQUID cohort study of almost 4000 older adults in southwest France found that levels of aluminum consumption in drinking water in excess of 0.1 mg per day were associated with a doubling of dementia risk and a three-fold increase in the risk of Alzheimer’s disease [33]. Several studies have shown that an elevated aluminum content could be detected in the brains of Alzheimer’s patients [29, 34, 35] and is often associated with NFTs, lipofuscin, and senile plaques [34]. There is considerable evidence that aluminum plays an important role in the dephosphorylation of tau proteins, development of NFTs, accumulation of amyloid beta protein, and formation of amyloid plaques [36–38]. Studies on occupational aluminum exposure have found similar results [18, 19, 39], and Yang et al. [19] observed that workers with occupational aluminum exposure showed significant decreases in global DNA methylation with an increase in serum aluminum concentration. Although the epidemiological evidence is inconsistent, it cannot be denied that the weight of evidence implicating aluminum in the causation of Alzheimer’s in at least some patients is increasing.

This study had some limitations. First, the association between aluminum exposure and cognitive impairment was not proof of the cause of one by the other. Second, our study was limited by the lack of measurements of aluminum concentration in blood and urine samples. Thus, biochemical investigation is called for in future studies. Third, the survey lacked information about neurological disease.

Aluminum production is an important industry in China. In 2017, China’s bauxite production ranked second worldwide. Thus, more attention needs to be paid to the risk of aluminum exposure among workers in factories and residents of the surrounding areas, including the possible risk of consequent cognitive impairment. Further research is warranted to establish a causal link between aluminum exposure and cognitive impairment.
